# Bayesian spatio-temporal analysis of dengue transmission in Lao PDR

**DOI:** 10.1038/s41598-024-71807-3

**Published:** 2024-09-12

**Authors:** Mick Soukavong, Kavin Thinkhamrop, Khanittha Pratumchart, Chanthavy Soulaphy, Phonepadith Xangsayarath, Mayfong Mayxay, Sysavanh Phommachanh, Matthew Kelly, Kinley Wangdi, Archie C. A. Clements, Apiporn T. Suwannatrai

**Affiliations:** 1 Doctor of Public Health Program, Faculty of Public Health, Khon Kaen University, Khon Kaen, Thailand; 2 Department of Parasitology, Faculty of Medicine, Khon Kaen University, Khon Kaen, Thailand; 3 National Center for Laboratory and Epidemiology (NCLE), Ministry of Health, Vientiane, Lao People’s Democratic Republic; 4 Lao-Oxford-Mahosot Hospital-Wellcome Trust Research Unit, Microbiology Laboratory, Mahosot Hospital, Vientiane, Lao People’s Democratic Republic; 5 Institute of Research and Education Development, University of Health Sciences, Vientiane, Lao People’s Democratic Republic; 6 Centre for Tropical Medicine and Global Health, Nuffield Department of Medicine, Oxford University, Oxford, UK; 7 Saw Hwee Hock School of Public Health, National University of Singapore, Singapore, Singapore; 8 National Centre for Epidemiology and Population Health, College of Health and Medicine, Australian National University, Canberra, Australia; 9 HEAL Global Research Centre, Health Research Institute, Faculty of Health, University of Canberra, Canberra, Australia; 10 Queen’s University Belfast, Belfast, Northern Ireland UK

**Keywords:** Dengue, Zoonotic disease, Temporal, Spatial, Bayesian, Lao PDR, Ecology, Microbiology, Diseases, Medical research

## Abstract

Dengue, a zoonotic viral disease transmitted by *Aedes* mosquitoes, poses a significant public health concern throughout the Lao People’s Democratic Republic (Lao PDR). This study aimed to describe spatial–temporal patterns and quantify the effects of environmental and climate variables on dengue transmission at the district level. The dengue data from 2015 to 2020 across 148 districts of Lao PDR were obtained from the Lao PDR National Center for Laboratory and Epidemiology (NCLE). The association between monthly dengue occurrences and environmental and climate variations was investigated using a multivariable Zero-inflated Poisson regression model developed in a Bayesian framework. The study analyzed a total of 72,471 dengue cases with an incidence rate of 174 per 100,000 population. Each year, incidence peaked from June to September and a large spike was observed in 2019. The Bayesian spatio-temporal model revealed a 9.1% decrease (95% credible interval [CrI] 8.9%, 9.2%) in dengue incidence for a 0.1 unit increase in monthly normalized difference vegetation index at a 1-month lag and a 5.7% decrease (95% CrI 5.3%, 6.2%) for a 1 cm increase in monthly precipitation at a 6-month lag. Conversely, dengue incidence increased by 43% (95% CrI 41%, 45%) for a 1 °C increase in monthly mean temperature at a 3-month lag. After accounting for covariates, the most significant high-risk spatial clusters were detected in the southern regions of Lao PDR. Probability analysis highlighted elevated trends in 45 districts, emphasizing the importance of targeted control strategies in high-risk areas. This research underscores the impact of climate and environmental factors on dengue transmission, emphasizing the need for proactive public health interventions tailored to specific contexts in Lao PDR.

## Introduction

Dengue is a zoonotic disease caused by a virus belonging to the Flaviviridae Family. The pathogen, Dengue virus (DENV), comprises four distinct serotypes: DENV-1, DENV-2, DENV-3, and DENV-4. Transmission primarily occurs through the bite of infected female *Aedes* mosquitoes. Endemic in tropical and subtropical regions, dengue stands as the fastest-growing arboviral disease globally^1^. DENV infection can lead to a spectrum of pathological conditions, spanning from mild and asymptomatic dengue fever (DF) to severe and potentially fatal conditions such as dengue hemorrhagic fever (DHF) and dengue shock syndrome (DSS)^2^. The global spread of DENV has undergone a dramatic expansion, propelled by factors such as rapid urbanization, heightened international travel, inadequate mosquito control, and the forces of globalization^3^. Tropical and sub-tropical countries, particularly those in the Western Pacific region and Southeast Asia, experience notably high incidences of dengue infection^4^. On a global scale, the yearly count of new dengue cases ranges from 50 to 100 million, leading to around 500,000 hospitalizations annually due to severe dengue, accompanied by a case fatality rate of 2.5%^5,6^. Worldwide, over two billion individuals face the risk of dengue infection, primarily concentrated in Asian nations, among them the Lao People’s Democratic Republic (Lao PDR) ^7^. In the Southeast Asia, between 2001 and 2010, the yearly hospitalization count for dengue patients averaged around 816,000, resulting in 5900 fatalities, while Lao PDR accounted for 18,000 cases and 41 deaths^8^.

In June 2019, the World Health Organization (WHO) highlighted a significant surge in dengue cases compared to the corresponding period of the prior year in Asian countries, including Lao PDR, Cambodia, Vietnam, Philippines, Malaysia, and Bangladesh. Dengue has evolved into a significant and pressing public health concern within Lao PDR^9^. Historical dengue epidemics have occurred in the past, with notable instances including 17,500 cases in 2003, 22,890 cases with 46 fatalities in 2010, and a higher incidence of 44,171 cases along with 95 deaths recorded in 2013^10^. In the southern region of the country alone, there were 4638 reported cases and 32 deaths^10^. Additionally, there have been shifts in dominant dengue virus serotypes over the years. For example, the major serotypes included DENV-2 and DENV-3^11^, with DENV-3 being predominant in 2013 and DENV-1 in 2015^12^. In 2019, a significant outbreak recorded 39,091 cases and 76 deaths, with DENV-2 as the predominant serotype among the specimens collected^13^.

Environmental and climate variables serve as predictive factors for dengue infection^14^. Seasonal climate variations correlate with *Aedes aegypti* abundance and historical dengue incidence^15^. Temperature influences mosquito dynamics, affecting larval development and transmission potential^16,17^. The interplay between rainfall and dengue is intricate, as rainfall can create breeding sites for the vector^18^ but excessive rain can also eliminate these sites^18,19^. In the dry season, augmented household water storage might diminish or even invert the positive correlation between dengue and rainfall^20,21^. Additionally, the rising urban population percentages have been significantly linked to an increased risk of dengue fever^22^. Other environmental factors like normalized difference vegetation index (NDVI) has been found to have a negative association with dengue incidence in many studies^23–25^. Seasonal patterns of dengue cases varied across different regions due to differential climatic and environmental factors. For example, Northern and Northeastern Thailand consistently saw peak cases from June to August^26^. While, Vietnam experienced recurrent seasonal epidemics with interruptions every three years, peaking from July to October^27^. Cambodia’s dengue transmission occurred mainly during the wet season (May to October)^28^.

Therefore, gaining insights into the local spatial and temporal dynamics of dengue, along with identifying the correlation between dengue occurrence and environmental and climatic factors, is indispensable for precision in targeting surveillance and control initiatives within a specific region. In recent times, Bayesian techniques have found application in analyzing the spatio-temporal distribution of dengue transmission. These methods offer the flexibility to integrate prior knowledge, including insights into the spatial and temporal characteristics of the data, while also allowing the concurrent modeling of spatial and temporal correlations alongside the influence of covariates^29^. Thus, the aim of this research was to analyze the spatial and temporal patterns of dengue in Lao PDR and assess the influence of local climate and environmental factors on the transmission of dengue fever.

## Materials and methods

### Study setting

**Fig. 1:**
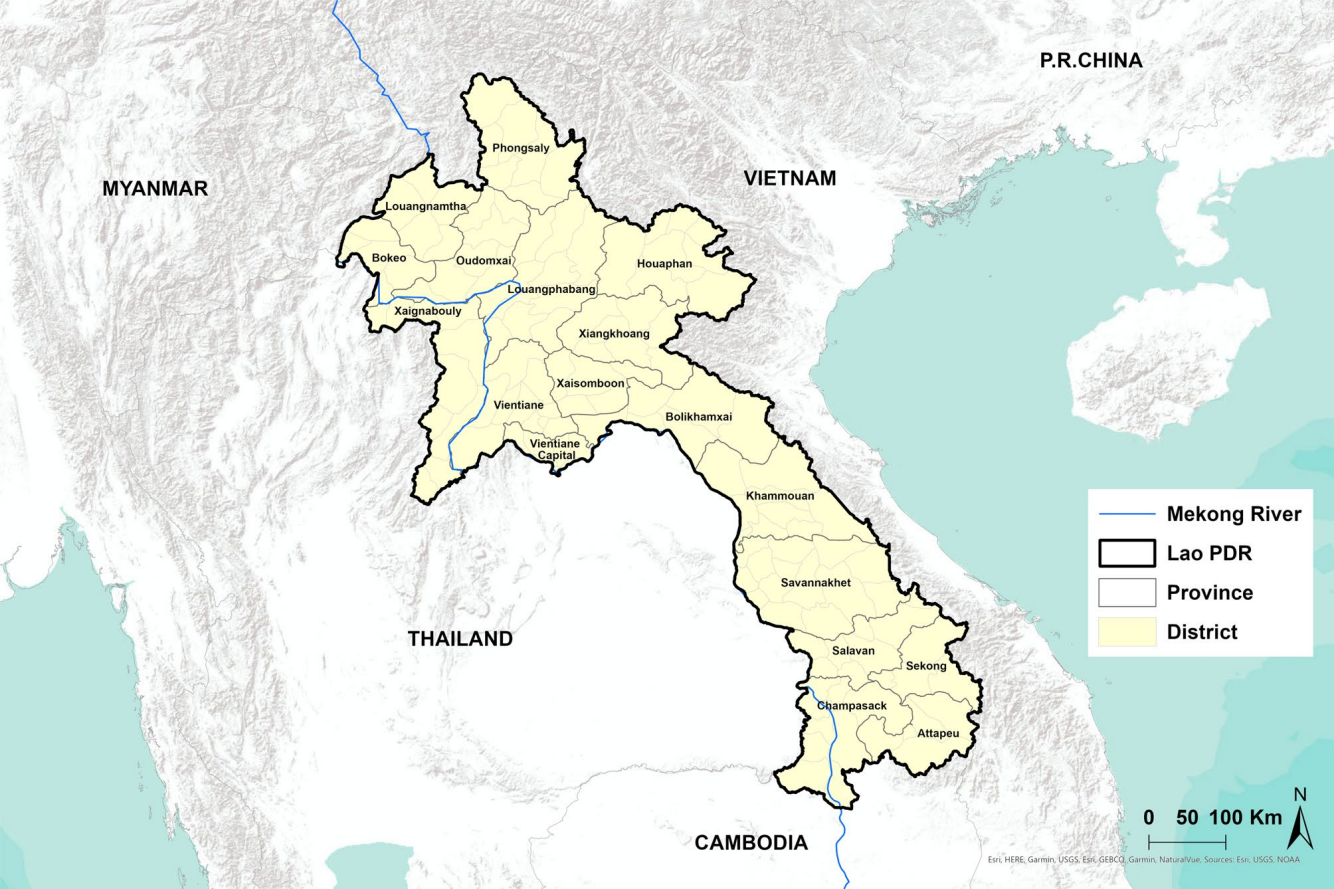
Map of Lao PDR and neighboring countries.

The research was conducted within Lao PDR, which spans an area of approximately 236,800 km^2^, situated at coordinates 18° 0′ 0″ N, 105° 0′ 0″ E (18, 105). Lao PDR is positioned in Southeastern Asia. The country is landlocked and is bordered by Thailand to the southwest, Vietnam to the east, Cambodia to the south, and China and Myanmar to the north. It comprises 18 provinces and 148 districts (Fig. 1). As of 2020, the population stands at 7,231,000, with the majority (68%) residing in rural areas; however, urbanization is progressing at a rate of 4.9% annually. The terrain is predominantly mountainous, with the most fertile land located along the Mekong plains. This river flows southward, forming more than 60% of the border with Thailand^30^.

Lao PDR exhibits a tropical climate, strongly influenced by the southeast monsoon that contributes 70% of the yearly precipitation, coupled with high humidity. Two distinct seasons define the climate: the rainy season, spanning May to mid-October, and the dry season, from mid-October to April. Annual average rainfall can reach 3000 mm (mm). The mean annual temperatures differ across regions, with northern and eastern mountainous areas and plateaus observing temperatures around 20 °C, while the plains experience higher temperatures ranging between 25 and 27 °C.

### Data collection

#### Dengue case data

We utilized dengue data obtained from case reports sourced from the National Center for Laboratory and Epidemiology (NCLE) in Lao PDR. Monthly dengue cases were documented in 148 districts of Lao PDR from January 2015 to December 2020. Since 1998, dengue has been designated as a nationally reportable disease by the NCLE in Lao PDR. In this reporting framework, epidemiologists from district hospitals collect and submit aggregated data to the Department of Health daily. Subsequently, the Department of Health consolidates this information and conveys it to the NCLE weekly^31^.

#### Population and administrative data

We obtained district level population data for the years 2015–2020 from the annually updated population projections from the Lao Statistics Bureau^32^. The geographic boundaries defining the 148 districts within Lao PDR were acquired from the administrative demarcations available on the DIVA-GIS website (www.diva-gis.org).

#### Environment and climate and data

Environmental and climatic data spanning the period from January 1, 2015, to December 31, 2020, were obtained at a monthly temporal resolution for the 148 districts of Lao PDR. The Google Earth Engine (GEE) platform was employed to access and process the following variables: monthly mean temperature (TEMP) and normalized difference vegetation index (NDVI). Additionally, precipitation (PREC) data were acquired from the Center for Hydrometeorology and Remote Sensing, while altitude (ALT) data were obtained from the WorldClim database (Table 1).

**Table 1:** Climate and environmental variables, Lao PDR, 2015–2020.

Variables	Detail	Spatial resolution	Source	Dataset name
ALT (masl)		0.86 km^2^	WorldClim	
NDVI (unit)	Monthly average	30 m and 10 m	Google Earth Engine	LANDSAT/LC08/C01/T1 and COPERNICUS/S2
PREC (mm)	Monthly average	4 km^2^	Center for Hydrometeorology and Remote Sensing	PERSIANN CCS
TEMP (°C)	Monthly average	9 km^2^	Google Earth Engine	ECMWF/ERA5_LAND/MONTHLY

*ALT* altitude, *NDVI* normalized difference vegetation index, *PREC* precipitation, *TEMP* mean temperature, *masl* meters above sea level, *mm* millimeter.

### Analysis

#### Dengue incidence

The average annual dengue incidence per 100,000 residents for the years 2015 to 2020 in Lao PDR was determined. The calculation involved dividing the average reported dengue cases from 2015 to 2020 by the mean population of the respective districts.

#### Crude standardized morbidity ratios

For the purpose of conducting an initial descriptive analysis of dengue incidence, we computed crude standardized morbidity ratios (SMR) for every district utilizing the subsequent formula:

$${Y}_{i}= \frac{{O}_{i}}{{E}_{i}}$$

Within this framework, *Y*_*i*_ denotes the comprehensive SMR within *i*-th district*.* The collective count of documented dengue cases within the district is designated as *O*_*i*_*,* whereas *E*_*i*_ represents the expected number of dengue cases in the *i*-th district. The expected number of cases was computed by multiplying the national dengue incidence rate by the mean population of each district during the study period.

### Exploration of seasonal patterns and temporal trends

The monthly numbers of dengue cases were calculated for the full-time series from January 2015 to December 2020. The time series was then decomposed into three temporal components using locally-weighted regression or loss: seasonality, trend, and residual variability. This decomposition method separates the time series into its seasonal pattern (*S*_*t*_), temporal trend (*T*_*t*_), and residual variability (*R*_*t*_). The original time series data, the seasonal component, trend component, and remainder component are denoted as *Y*_*t*_*, S*_*t*_*, T*_*t*_*,* and *R*_*t*_ respectively, for each month *t* ranging from 1 to N.

$${Y}_{t}={S}_{t}+{T}_{t}+{R}_{t}$$

The time series data was decomposed, and all the mentioned parameters were extracted using R Studio with the function *stl*, and the parameter setting "periodic" applied^33^. In the final model, a logarithmic transformation was employed to evaluate the significance level of the trend^34^.

### Spatial autocorrelation

Moran’s I statistic was employed to assess the presence and strength of spatial autocorrelation across the entire study area. This statistic also served to test the assumption of spatial independence during the implementation of spatial pattern analysis^35^. The Local Indicators of Spatial Association (LISA) technique including Anselin Local Moran’s I statistic and Getis-Ord Gi* statistics were utilized. These methods allowed for the detection of spatial clusters and areas of high concentration^35^. These analyses were conducted using tools provided in ArcGIS Pro version 3.2 software (ESRI, Redlands, CA)^36^.

### Spatio-temporal models

An initial univariate Poisson regression was conducted to select covariates for the study. The dependent variable was the number of dengue cases, while the independent variables included various environmental and climatic variables (ALT, NDVI, TEMP, and PREC) without a lag and with 1, 2, 3, 4, 5, and 6-month lag times. The independent variables selected based on the lowest values of the Akaike’s information criterion (AIC), Bayesian information criterion (BIC), and a significant Incidence Rate Ratio (IRR) with a p-value < 0.05 were selected^37^ (Supplementary Table 1). To assess collinearity, Pearson correlation analysis was performed on all the included variables. The collinearity of the covariates was also tested using the variance inflation factor (VIF) statistic as a diagnostic tool where, covariates with a VIF > 4.0 were considered to be collinear and removed from the model ^38^ (Supplementary Table 2).

Zero-inflated Poisson (ZIP) regression showed a better fit over standard Poisson regression with lower AIC and BIC. A significant difference was observed between the two models as demonstrated by the Vuong test (Supplementary Table 3). ZIP regression with four models was constructed in a Bayesian framework. Model I incorporated only independent variables as explanatory factors. Model II introduced spatially structured random effects in addition to the independent variables. Model III encompassed both independent variables, spatially structured random effects, and unstructured random effects. Model IV was same as Model III but included a spatio-temporal random effect to estimate spatial variability in district temporal trends. In the last model, dengue incidence *Y*, for *i*-th district (*i* = 1, …, 148) in the *j*-th month (*j* = 1, …, 72; January 2015–December 2020) was structured as follows:

$$P(Y_{ij} = y_{ij} ) = \left\{ {\begin{array}{*{20}l} {\omega + 1(1 - \omega )e^{ - \mu } ,} \hfill & {y_{ij} = 0} \hfill \\ {(1 - \omega )e^{ - \mu } \mu_{ij}^{{y_{ij} }} /y_{ij} ,} \hfill & {y_{ij} > 0;} \hfill \\ \end{array} } \right.$$

$$\begin{aligned} & Y_{ij} \sim {\text{Poisson}}(\mu_{ij} ) \\ & {\text{log}}(\mu_{ij} ) = {\text{log}}({\text{E}}_{ij} ) + \theta_{ij} \\ & \theta_{ij} = \alpha + \beta_{1} \times {\text{trend}}_{j} + \beta_{2} \times {\text{ALT}} + \beta_{3} \times {\text{NDVI}} + \beta_{{4}} \times {\text{PREC}} + \beta_{{5}} \times {\text{TEMP}} + u_{i } + v_{ij} + w_{ij} \\ \end{aligned}$$

where *E*_*ij*_ is the expected number of cases (acting as an offset to control for population size) in district *i* and *j* month; *θ*_*ij*_ is the mean log relative risk (RR); α is the intercept, and *β*_*1*_*, β*_*2*_*, β*_*3*_*, β*_*4,*_ and *β*_*5*_ are the coefficients for the overall temporal trend of dengue risk, ALT, NDVI with a 1-month lag, PREC with a 6-month lag and TEMP with a 3-month lag respectively; u_*i*_ is the unstructured random effect with mean zero and variance σ_u_^2^ and v_*i*_ is the spatially structured random effect with mean zero and variance σ_v_^2^ and w_*ij*_ is the spatio-temporal random effect with a mean of zero and variance of σ_w_^2^.

A conditional autoregressive (CAR) prior structure was employed to model both the spatially structured random effect and the spatio-temporal random effect, allowing for smoothing of the district-level temporal trends. Spatial relationships between the districts were determined using an adjacency weights matrix, where a weight of 1 was assigned if two districts shared a border, and 0 if they did not. The intercept was assigned a flat prior distribution, while the coefficients followed a normal prior distribution (with a mean of 0 and precision, the inverse of variance, set at 0.00001). Non-informative gamma distributions with shape and scale parameters equal to 0.001 were used to specify the priors for the precision of the unstructured and spatially structured random effects, as well as the spatio-temporal random effects.

To ensure convergence, an initial burn-in period of 10,000 iterations was executed, and the results were discarded. Convergence was evaluated by visually examining posterior density and history plots. Convergence was observed at approximately 100,000 iterations for each model. The posterior distributions of each model parameter were stored and summarized, including the posterior mean and 95% credible intervals (CrI). The deviance information criterion (DIC) was computed to facilitate model selection, with a lower DIC indicating a better fit of the model. A significance level (α) of 0.05 was employed in all analyses to determine statistical significance. Univariate regression analysis was performed using Stata statistical software (version 14; Stata Corp, College Station, Texas, USA)^39^. The Bayesian models were developed using the WinBugs statistical software version 1.4.3 (Medical Research Council, Cambridge, UK)^40^. Chloropleth maps of SMR and the spatio-temporal random effects were produced using ArcGIS Pro version 3.2 software (ESRI, Redlands, CA).

## Results

### Descriptive statistics

**Fig. 2:**
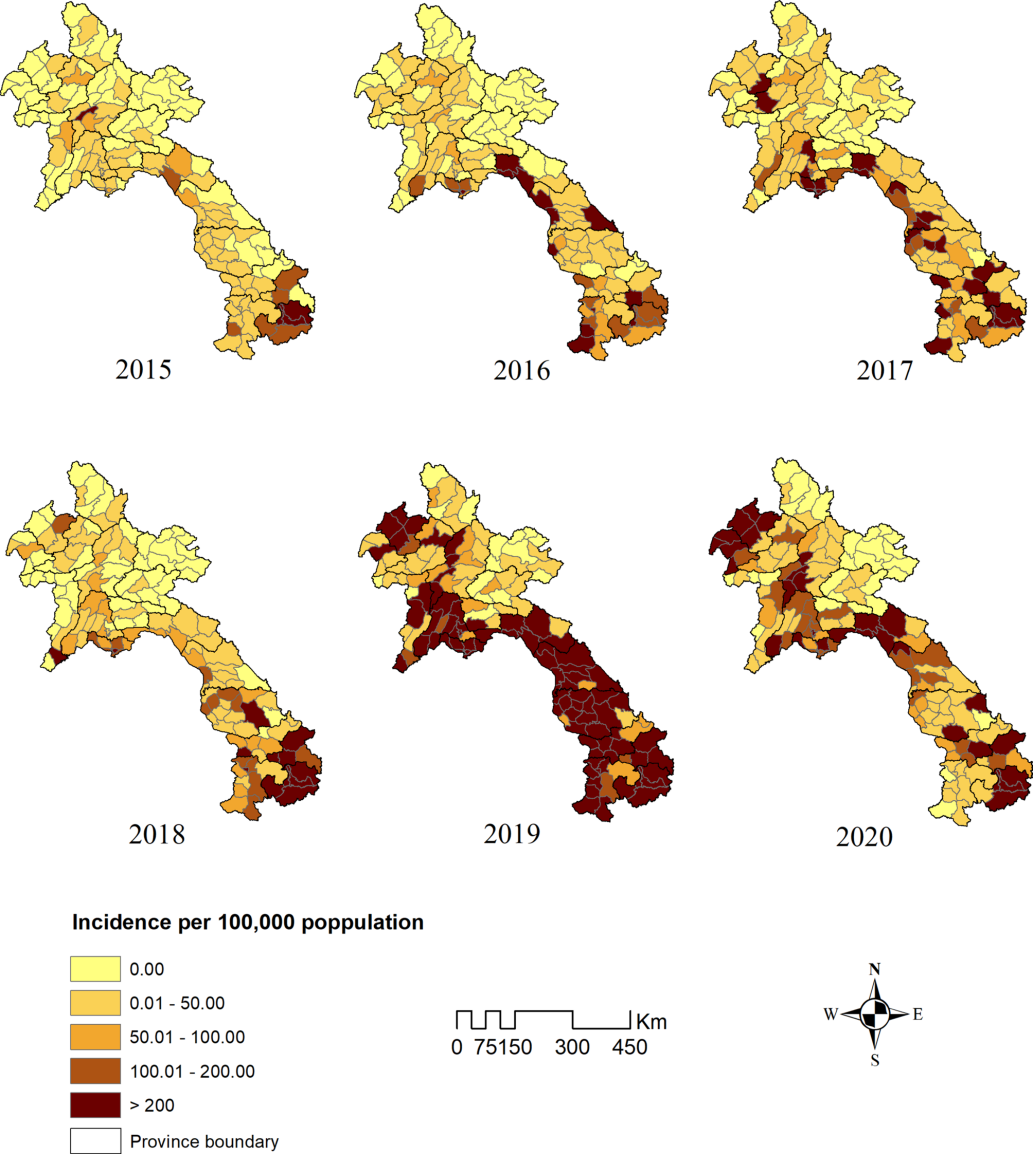
Dengue incidence rates by districts, Lao PDR, year 2015–2020.

During the period from January 2015 to December 2020, a total of 72,471 dengue cases were reported to the NCLE. The incidence rate of dengue was calculated at 174 per 100,000 inhabitants. The distribution of dengue exhibited variation among districts, with incidence rates ranging from zero to exceeding 200 per 100,000 population each year throughout the study period (Fig. 2).

The median ALT in Lao PDR, measured in meters above sea level (masl) was 648.20 (interquartile range [IQR]: 258.23–858.00 masl), ranging from 136.00 to 1333.60 masl. NDVI has a median value of 0.3 2 (IQR: 0.17–0.47 unit). PREC, measured in millimeters (mm) had a median of 55.37 (IQR: 3.64–198.05 mm), ranging from 0 to 1733.07 mm. TEMP, measured in degree Celsius (°C), had a median of 23.71 °C (IQR: 21.48–25.63 °C) ranging from 12.11 to 32.24 °C (Table 2).

**Table 2:** Distribution of monthly means of climate and environmental variables, Lao PDR, 2015–2020.

Variables	Median (IQR)	Min–Max
ALT (masl)	648.20 (258.23–858.00)	136.00–1333.60
NDVI (unit)	0.32 (0.17–0.47)	− 0.03 to 0.77
PREC (mm)	55.37 (3.64–198.05)	0.00–1733.07
TEMP (°C)	23.71 (21.48–25.63)	12.11–32.24

*ALT* altitude, *NDVI* normalized difference vegetation index *PREC* precipitation, *TEMP* mean temperature, *IQR* interquartile range, *masl* meters above sea level, *mm* millimeter.

**Fig. 3:**
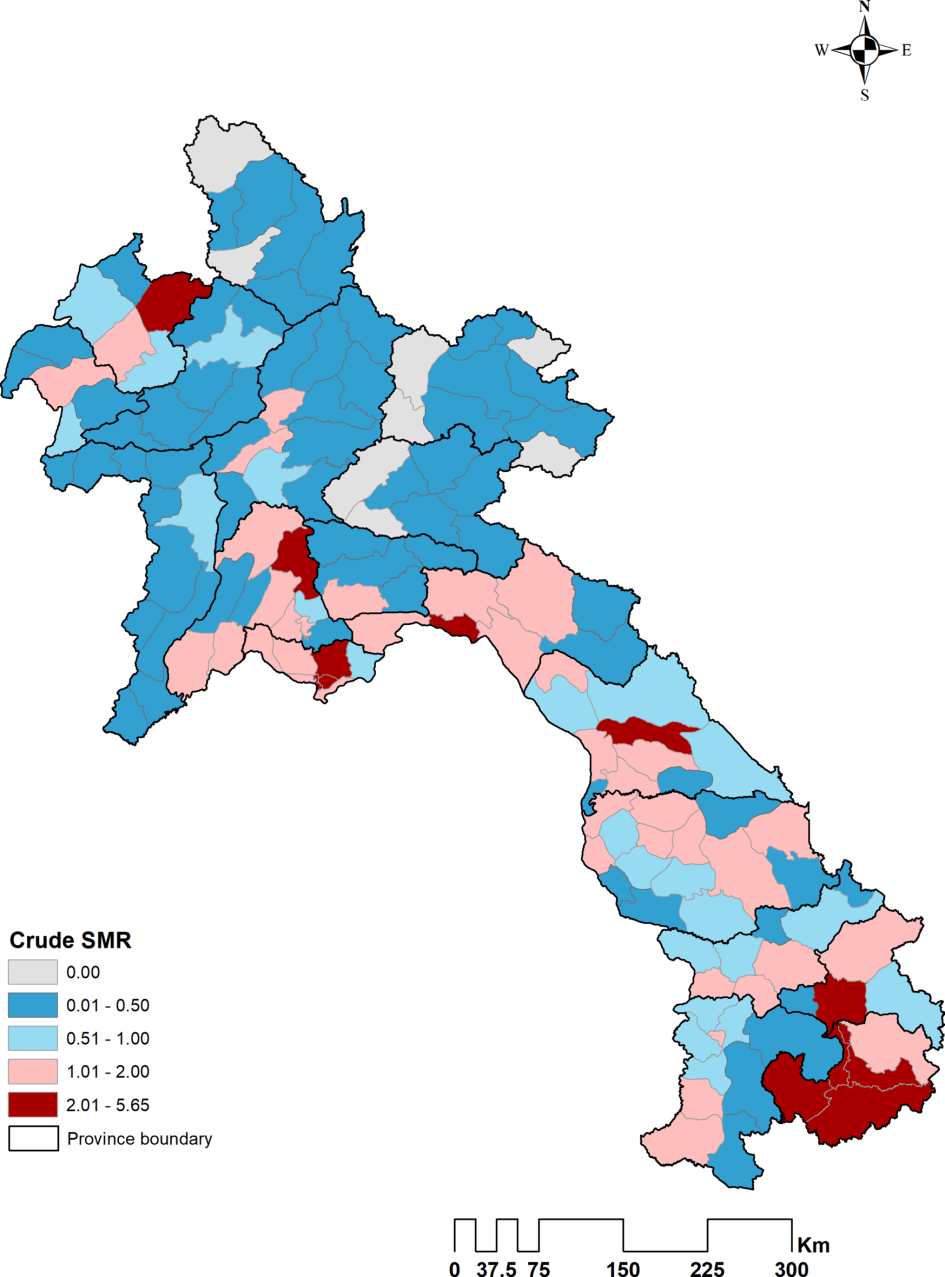
Crude standardized morbidity ratios (SMR) of dengue by districts in Lao PDR, year 2015–2020.

There was significant spatial variation in the SMR for dengue throughout the study period, with a concentration of higher SMR values, surpassing 2.01, notably observed in specific districts. In Attapeu Province, the districts of Xaysetha, Samakkhixay, Sanamxay, and Phouvong exhibited particularly high SMR values. Similarly, in Sekong Province, the district of Lamarn recorded an elevated SMR. Additionally, Khammouan Province, the district of Nhommalath, in Bolikhamxai Province, the district of Pakxane, and in Louangnamtha Province, the district of Namtha, also showed high SMR values. Notably, districts in Vientiane Capital, including Xaysetha, Chanthabuly, Sisattanak, and Xaythany, consistently displayed high SMR values (Fig. 3).

### Time series decomposition

**Fig. 4:**
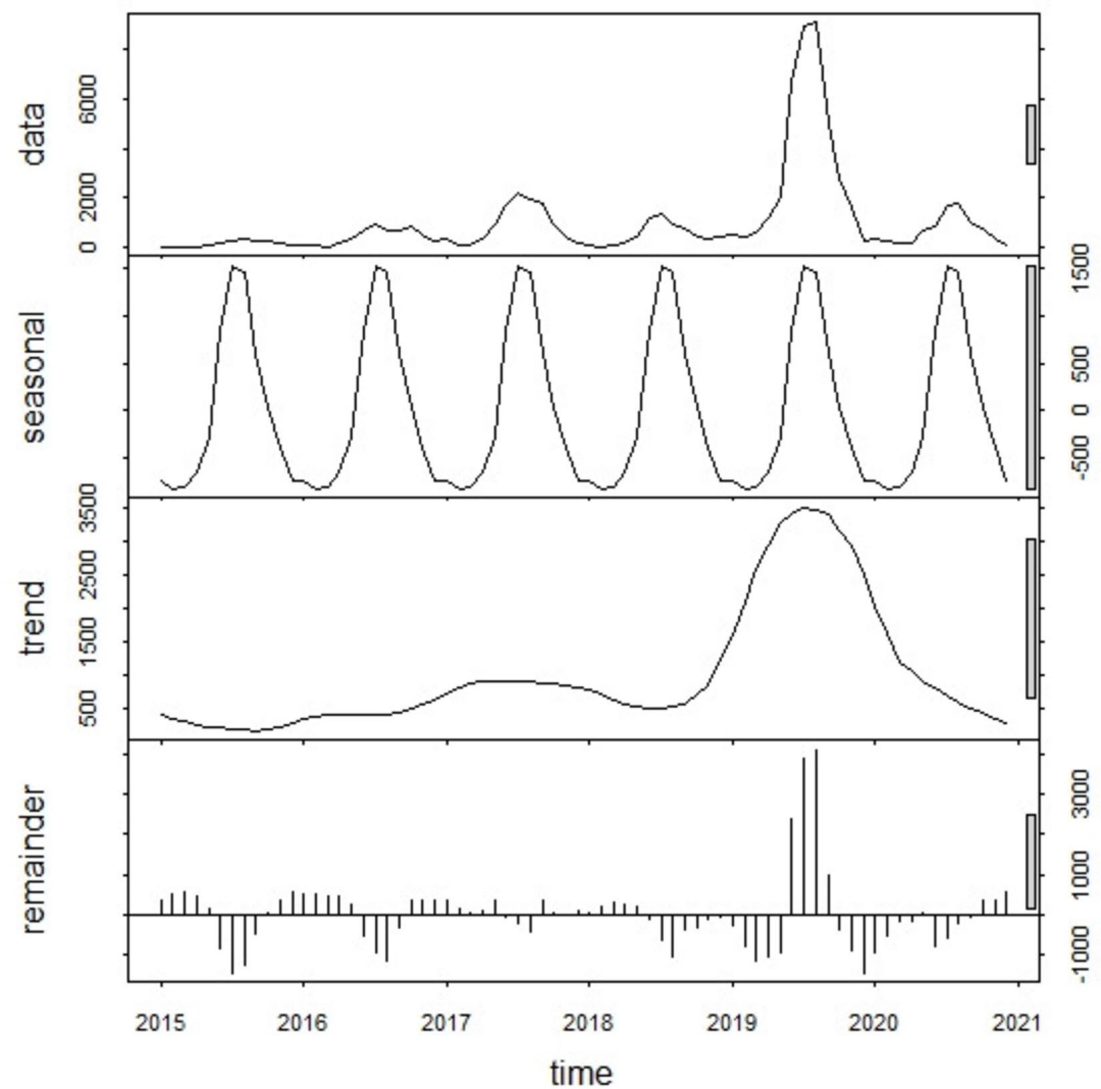
Temporal decomposition of numbers of dengue cases of Lao PDR, year 2015–2020.

The seasonal decomposition plot demonstrated strong seasonality with a peak occurring between June and September each year. The inter-annual pattern showed a large peak in 2019.The residuals in the fourth panel showed elevated variability in the years 2019 (Fig. 4).

### Spatial autocorrelation analysis

**Fig. 5:**
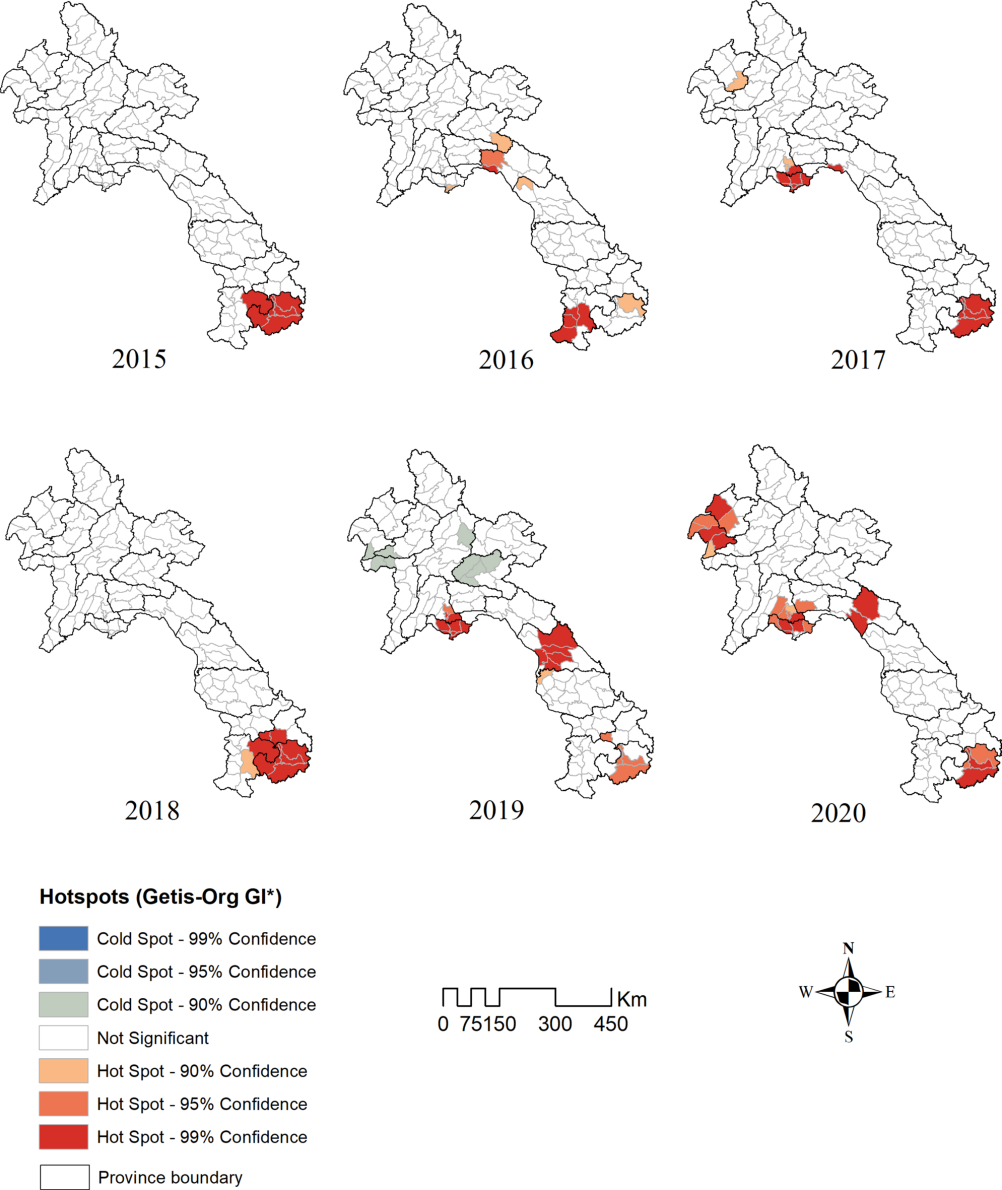
Spatial hotspots of dengue in Lao PDR, year 2015–2020, based on the Getis-Ord statistic in each year (2015–2020).

The global Moran’s I statistic for the dengue cases is each year between 2015 and 2020 were 0.40, 0.28, 0.74, 0.31, 0.47, and 0.27 respectively (p-value < 0.001), indicating the presence of significant, positive spatial autocorrelation of dengue over the whole study area. Hotspot analysis using the Getis-Ord statistic in each year (2015–2020) showed that a significant hotspots district mostly located in Attapeu, Vientiane Capital and Champasack provinces (Fig. 5). In addition, cluster analysis using LISA showed 5, 9, 9,7, 13, and 14 high-high clusters in 2015–2020 respectively (Supplementary Fig. 1), also located mostly in and around Vientiane Capital, Champasack and Attapeu provinces. However, in 2020 some districts in the Northern part of the country including Louangnamtha and Bokeo provinces showed significant hotspots and high-high clusters.

### Spatio-temporal model

**Fig. 6:**
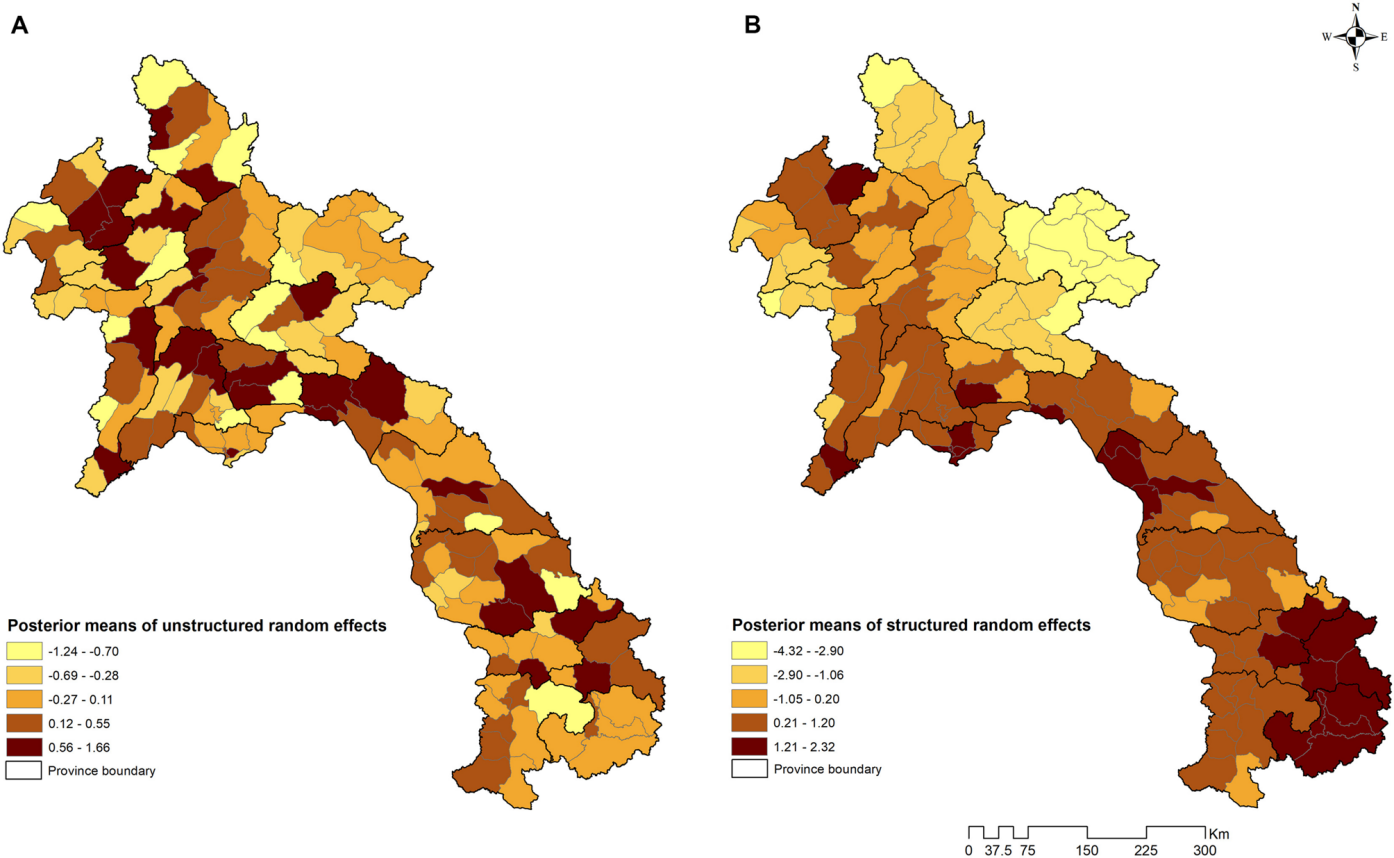
Spatial distribution of posterior means of (**A**) unstructured and (**B**) structured random effects of dengue in Lao PDR, year 2015–2020.

**Fig. 7:**
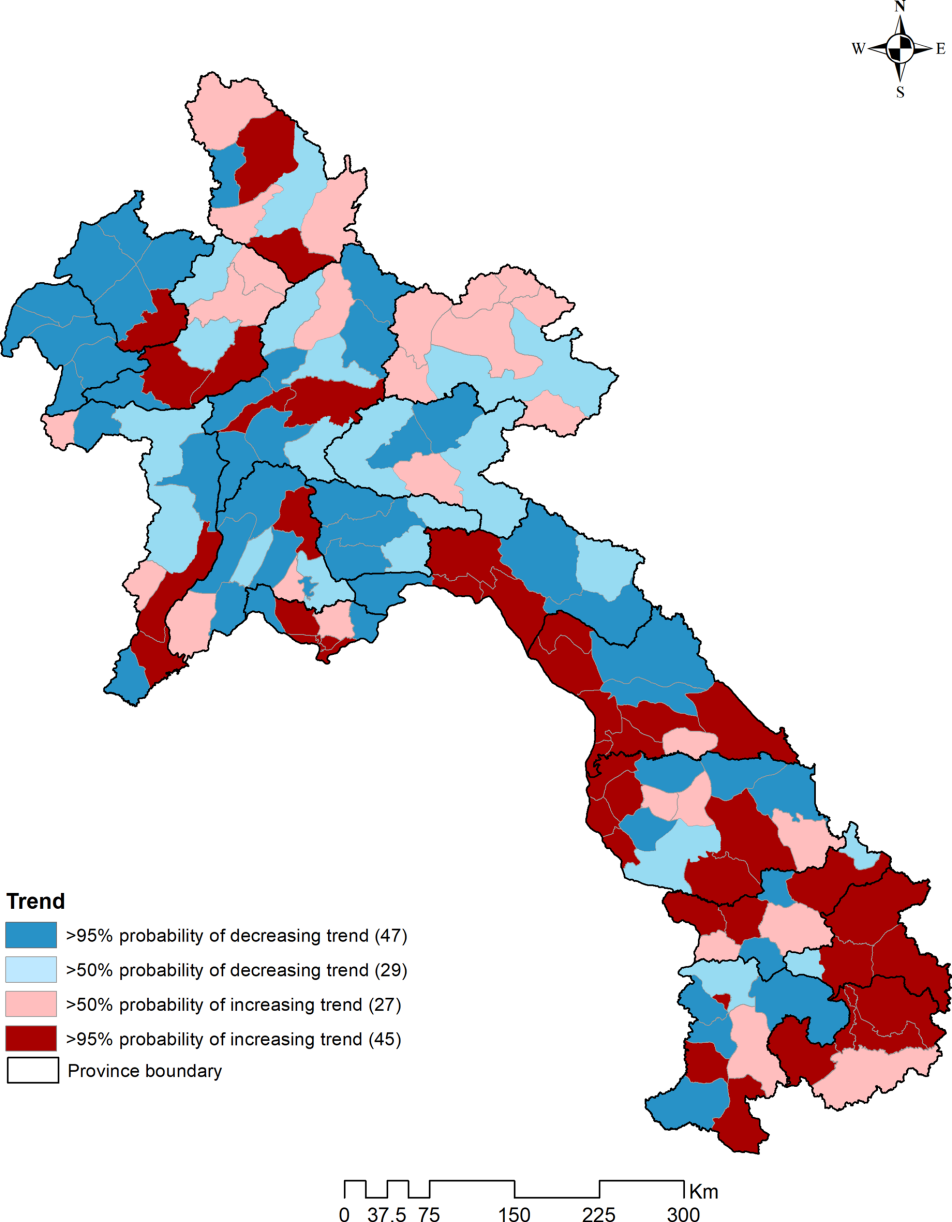
Trend analysis of dengue in Lao PDR, year 2015–2020.

We selected Model IV, which incorporates unstructured, structured, and spatio-temporal random effects, as it exhibited the lowest DIC. Dengue incidence exhibited a 9.1% decrease (95% CrI 8.9%, 9.2%) for a 0.1 unit increase in NDVI at a 1-month lag. Furthermore, there was a decrease of 5.7% (95% CrI 5.3%, 6.2%) in dengue incidence for a 1 cm increase in PREC at a 6-month lag. In contrast, dengue incidence showed an increase of 43% (95% CrI 41%, 45%) for a 1 °C rise in mean TEMP at a 3-month lag (Table 3). The maps of the posterior means of the spatially structured random effect showed most higher mean dengue risk in the southern part of the country after accounting for the covariates (Fig. 6). Spatial clustering was clearly observable in Attapeu and Sekong provinces, as well as in certain districts of Champasack and Salavan provinces. A > 95% probability of a higher than national average trend was observed in 45 districts, mostly situated in the Southern and Central regions, constituting 48.2% in the southern part and 44.2% of the total in the Middle part. At the provincial level, the proportion of number of districts were as follows: Attapeu 80%, Sekong 75%, Khammoun 70%, Vientiane Capital 66.7%, and Bolikhamxai 42.9%. These suggest a significant increase in the incidence of dengue in this region of the country (Fig. 7).

**Table 3:** Parameter estimates from Bayesian Zero-Inflated Poisson regression models of dengue cases in Lao PDR, year 2015–2020.

Variables	Model I	Model II	Model III	Model IV
	RR (95% CrI)	RR (95% CrI)	RR (95% CrI)	RR (95% CrI)
Alpha*	− 1.19 (− 1.35 to − 1.04)	− 1.30 (− 1.35 to − 1.25)	− 1.31 (− 1.48 to − 1.15)	− 1.57 (− 1.80 to − 1.33)
Mean monthly trend	1.35 (1.34–1.36)	1.35 (1.32–1.38)	1.35 (1.33–1.37)	1.59 (1.52–1.67)
ALT (masl)	1.00 (0.20–4.83)	1.00 (0.21–4.88)	1.00 (0.21–4.87)	1.00 (0.21–4.83)
NDVI with 1-month lag (unit)	0.11 (0.10–0.12)	0.11 (0.09–0.14)	0.11 (0.09–0.13)	0.09 (0.08–0.11)
PREC with 6-month lag (mm)	0.994 (0.994–0.995)	0.994 (0.994–0.995)	0.994 (0.994–0.995)	0.994 (0.994–0.995)
TEMP with 3-month lag (°C)	1.42 (1.42–1.43)	1.42 (1.40–1.45)	1.42 (1.40–1.44)	1.43 (1.41–1.45)
Probability of extra zero	1.55 (1.54–1.57)	1.55 (1.53–1.57)	1.55 (1.53–1.57)	1.49 (1.47–1.51)
Heterogeneity*				
Unstructured	0.40 (0.32–0.50)	–	1.87 (1.07–3.07)	1.46 (0.77–2.47)
Structured	–	0.20 (0.16–0.24)	0.38 (0.24–0.59)	0.31 (0.20–0.47)
Structured (trend)				0.51 (0.39–0.63)
DIC	115,498	115,636	115,584	110,201

*RR* relative risk, *CrI* credible interval, *DIC* deviance information criterion, *ALT* altitude, *NDVI* normalized difference vegetation index, *PREC* precipitation, *TEMP* mean temperature. *Coefficient.

## Discussion

The study observed that dengue cases peaked each year between June and September. A spatio-temporal model revealed significant associations between environmental factors and dengue incidence, including decreases in incidence with higher vegetation index and precipitation, and increases with rising temperature. Spatial and temporal analyses identified high-risk areas particularly in the southern part of country. Probability analysis underscored elevated trends in 45 districts, highlighting the importance of targeted control strategies in these high-risk areas in Lao PDR.

Throughout the study period, a clear seasonal pattern was observed. The temporal analysis revealed a strong seasonality of dengue with a prominent peak occurring between June and September each year and a large peak in 2019. During the monsoon season, which typically spans from June to August, Lao PDR experiences heightened rainfall, increased humidity, and elevated temperatures. These climatic conditions create favorable environments for the breeding and proliferation of mosquitoes, particularly the *Aedes* mosquitoes responsible for transmitting the dengue virus^15–18^. The extended rainy period results in the accumulation of stagnant water in various containers, such as discarded tires, flowerpots, and other receptacles that serve as breeding grounds for mosquitoes. The higher humidity and temperatures further accelerate the mosquito’s life cycle, leading to a more rapid reproduction rate and an overall increase in the mosquito population^15–18^. That is consistent with the findings from studies in North and Northeast Thailand, Vietnam, Cambodia, and Bhutan, which led to a rise in dengue cases during this period^25–28,41^. Overall, the annual incidence of dengue varied in each year. However, in 2019, Lao PDR experienced a significant increase in dengue fever cases, with an incidence rate of 549 per 100,000 population. Similarly, Cambodia had very high incidence rates in same year, exceeding 600 cases per 100,000 population^42^. Furthermore, Thailand and Vietnam generally had higher incidence rates compared to Lao PDR during the period of our study. Thailand’s rates varied over the years, ranging between 82 and 223 cases per 100,000 population, often surpassing those of Lao PDR^43^. Vietnam’s rates consistently higher, ranging from 200 to 230 cases per 100,000 population^44^. In 2019, Lao PDR experienced a large-scale dengue fever outbreak due to the co-circulation of three DENV serotypes, an occurrence rare in the area. The main pathogenic agents of the outbreak were DENV-1 and DENV-2. This co-circulation likely contributed to more severe clinical symptoms and the expansion of the epidemic on a larger scale^45^. The southern belt regions exhibited a higher residual risk of dengue. These areas, characterized by elevated poverty levels and lower inequality, may indicate underlying factors predisposing individuals to increased dengue risk^46^. Moreover, these regions border Vietnam, Cambodia, and Thailand, potentially facilitating dengue transmission through cross-border human travel and trade^47^.

Dengue incidence exhibited a negative correlation with NDVI. Similar negative associations between NDVI and dengue incidence were documented in several other studies^24,25,48^. High NDVI values generally indicate sparsely populated areas that are unable to meet the conditions of human and mosquito contact to transmit dengue. Generally, higher dengue incidence is often associated with urban and peri-urban areas with increased human population density^49^. Such areas may have lower NDVI values due to urbanization, land use changes, and the replacement of natural vegetation with built environments. These urbanized settings are favorable for the *Aedes* mosquitoes, which transmit the dengue virus.

Our study found that a 1 cm increase in PREC with a 6-month lag significantly reduced the risk of dengue incidence by 0.57%. In the complex relationship between rainfall and dengue incidence, non-linearity is evident, primarily driven by rainfall’s impact on the adult female mosquito lifecycle. Increased rainfall fosters breeding habitats, resulting in a rise in vector populations. However, excessively high PREC levels may negatively affect mosquito density by washing out breeding sites^50^. The lag time between weather and dengue cases could be partly accounted for by the impact of weather conditions on the biological development of the mosquito vector including long egg-hatching periods and the high possibility of *Aedes’* eggs to survive waterless for several months^51^. Several studies reported a negative relationship between precipitation and dengue cases^27,52–54^. A study in India reported the relative risk of dengue gradually increases with cumulative weekly rainfall from 40 to 60 mm, but decreases when rainfall exceeds 80 mm^53^. The same as a study from Lao PDR, where a significant decrease in dengue risk was observed when heavy rainfall exceeded 200 mm^54^. Protective impacts on dengue incidence are observed with very high rainfall (> 70 mm) at a lag of 15–20 weeks and rainfall between 30 and 60 mm. This suggests a complex relationship between rainfall and dengue, with specific ranges act and thresholds influencing risk dynamics^55^.

This study demonstrated that a 43% increase in the risk of dengue incidence occurred with a 1 °C rise in mean temperature at 3-month lag. These positive relationship results were consistent with previous studies^28,52,56,57^. This can be attributed to various temperature-related factors influencing the dynamics of *Aedes* mosquitoes and the dengue virus. Optimal temperatures for DENV transmission generally fall within the range of 20–26 °C^58^. Temperature influences multiple aspects of mosquito biology, including egg-hatching rates, larval developmental time, and adult survival rates^28^. The result of a study from East Delhi, India showed that the model with a two-month lag provided the best prediction of dengue epidemics^59^, consistent with the study in Cambodia that the monthly average mean temperatures were significantly associated with dengue incidence at a lag of 0–3 months in three provinces^60^. This aligns with the known influence of temperature on mosquito population dynamics, affecting egg-laying, hatching, and the abundance of *Aedes* larvae and pupae^61^. Additionally, the higher temperatures also reduced the extrinsic incubation period (EIP) of dengue virus at 30 °C, which may facilitate the spread of dengue^59^, because of higher multiplication of viruses. This is also anticipated to promote the adoption of human behaviors that increase contact with disease vectors, such as spending extended periods outdoors or keeping windows open while indoors^62^.

This study has some inherent limitations. Firstly, the temperature effect could be influenced by the seasonality of dengue cases, which primarily occur during warmer months that are often preceded by other warm months. This potential connection between the temperature effect and seasonality is an interesting avenue to explore further. Secondly, essential information such as the diagnostic method, viral serotypes, patient age, and gender was unavailable, potentially leading to over- or under-reporting of cases. Lastly, the observed spatial and temporal variability may have been influenced by unaccounted socio-economic and ecological factors. Future research endeavors should incorporate these variables to comprehensively evaluate the genuine temporal relationship.

## Conclusion

In summary, our study revealed significant spatial variation in reported dengue infection, with a peak during the monsoon season and notable clusters around Vientiane Capital, Khammoun, and Attapeu provinces. The spatio-temporal model highlighted a decrease in dengue risk with increased NDVI and PREC but an increase with rising TEMP. The probability of above-average dengue trends was significant in the Southern and Middle regions, indicating a noteworthy increase. This emphasizes the rising trend of dengue with strong seasonality and highlights potential high-risk zones. The association with climatic factors supports the use of climate-based early warning systems for more effective control strategies in Lao PDR, advocating targeted resource allocation, including intensified surveillance and vector control in identified high-risk areas.

## Supplementary Information

Supplementary Table S1.

Supplementary Table S2.

Supplementary Table S3.

Supplementary Legend.

Supplementary Figure S1.

## Data Availability

The datasets generated during and/or analyzed for the current study will be made available from the corresponding author on reasonable request.
