# Supplementary material for: Bayesian spatio-temporal analysis of dengue transmission in Lao PDR
Source: Sci Rep. 2024 Sep 12;14:21327. doi: 10.1038/s41598-024-71807-3 (PMC11393087; doi:10.1038/s41598-024-71807-3)
Supplement: Supplementary file 1 — Supplementary Table S1. [file 41598_2024_71807_MOESM1_ESM.docx]

| **Variables** | **IRR** | **P-value** | **AIC** | **BIC** |
| --- | --- | --- | --- | --- |
| Altitude | 0.998718 | < 0.001 | 188873.1 | 188902.2 |
| Normalized difference vegetation index | |  |  |  |
| No lag | 0.076797 | < 0.001 | 184045.7 | 184074.8 |
| **One-month lag** | **0.070373** | **< 0.001** | **183948.1** | **183977.2** |
| Two-month lag | 0.125715 | < 0.001 | 189036.3 | 189065.4 |
| Three-month lag | 0.239109 | < 0.001 | 193279.7 | 193308.8 |
| Four-month lag | 0.413503 | < 0.001 | 195928.6 | 195957.7 |
| Five-month lag | 0.690534 | < 0.001 | 197301.6 | 197330.7 |
| Six-month lag | 1.202236 | < 0.001 | 197547.7 | 197576.8 |
| Precipitation |  |  |  |  |
| No lag | 1.001989 | < 0.001 | 186462.3 | 186491.4 |
| One-month lag | 1.002168 | < 0.001 | 186536.5 | 186565.6 |
| Two-month lag | 1.001766 | < 0.001 | 190722.8 | 190751.9 |
| Three-month lag | 1.000867 | < 0.001 | 196088.0 | 196117.1 |
| Four-month lag | 0.999095 | < 0.001 | 196537.5 | 196566.6 |
| Five-month lag | 0.997284 | < 0.001 | 190714.3 | 190743.4 |
| **Six-month lag** | **0.995838** | **< 0.001** | **185541.9** | **185571.0** |
| Temperature |  |  |  |  |
| No lag | 1.178626 | < 0.001 | 185749.3 | 185778.4 |
| One-month lag | 1.254603 | < 0.001 | 177550.5 | 177579.6 |
| Two-month lag | 1.335656 | < 0.001 | 166079.1 | 166108.2 |
| **Three-month lag** | **1.346101** | **< 0.001** | **162797.0** | **162826.1** |
| Four-month lag | 1.271796 | < 0.001 | 171713.9 | 171743.0 |
| Five-month lag | 1.126630 | < 0.001 | 188843.5 | 188872.6 |
| Six-month lag | 1.035573 | < 0.001 | 196171.6 | 196200.7 |
| *IRR* incidence rate ratio, *AIC* Akaike’s information criterion, *BIC* Bayesian information criterion. | | | | |

**Table S1.** Variable selection using Poisson regression, Akaike’s information criterion and Bayesian information criterion.
