# Supplementary material for: Bayesian spatio-temporal analysis of dengue transmission in Lao PDR
Source: Sci Rep. 2024 Sep 12;14:21327. doi: 10.1038/s41598-024-71807-3 (PMC11393087; doi:10.1038/s41598-024-71807-3)
Supplement: Supplementary file 2 — Supplementary Table S2. [file 41598_2024_71807_MOESM2_ESM.docx]

| **Variables** | **VIF** | **1/VIF** |
| --- | --- | --- |
| TEMP with 3-month lag (°C) | 2.04 | 0.49 |
| ALT (masl) | 1.69 | 0.59 |
| PREC with 6-month lag (mm) | 1.21 | 0.83 |
| NDVI with 1-month lag (unit) | 1.07 | 0.94 |
| Mean VIF | 1.50 |  |
| *VIF* variance inflation factor, *ALT* altitude, *NDVI* normalized difference vegetation index, *PREC* precipitation, *TEMP* mean temperature. | | |

**Table S2.** Variance inflation factor of the variables included in the model.
