# Supplementary material for: Bayesian spatio-temporal analysis of dengue transmission in Lao PDR
Source: Sci Rep. 2024 Sep 12;14:21327. doi: 10.1038/s41598-024-71807-3 (PMC11393087; doi:10.1038/s41598-024-71807-3)
Supplement: Supplementary file 3 — Supplementary Table S3. [file 41598_2024_71807_MOESM3_ESM.docx]

**Table S3.** Model comparison using Akaike’s information criterion and Bayesian information criterion.

| **Model** | **Observation** | **Number of parameters** | **AIC** | **BIC** | **Vuong's test p-value** |
| --- | --- | --- | --- | --- | --- |
| Poisson | 10,656 | 4 | 214587.5 | 214623.9 | < 0.001 |
| ZIP | 10,656 | 4 | 157384.7 | 157457.4 |  |
| *ZIP* Zero-inflated Poisson, *AIC* Akaike’s information criterion, *BIC* Bayesian information criterion | | | | | |
