# Supplementary material for: Bayesian spatio-temporal analysis of dengue transmission in Lao PDR
Source: Sci Rep. 2024 Sep 12;14:21327. doi: 10.1038/s41598-024-71807-3 (PMC11393087; doi:10.1038/s41598-024-71807-3)
Supplement: Supplementary file 4 — Supplementary Legend. [file 41598_2024_71807_MOESM4_ESM.docx]

**Supplementary Figures**

**Figure S1.** Spatial clustering of dengue in Lao PDR (year 2015 – 2020) based on the Getis-Ord Gi* statistics.
