# Supplementary figures and images for: Bayesian spatio-temporal analysis of dengue transmission in Lao PDR
Source: Sci Rep. 2024 Sep 12;14:21327. doi: 10.1038/s41598-024-71807-3 (PMC11393087; doi:10.1038/s41598-024-71807-3)

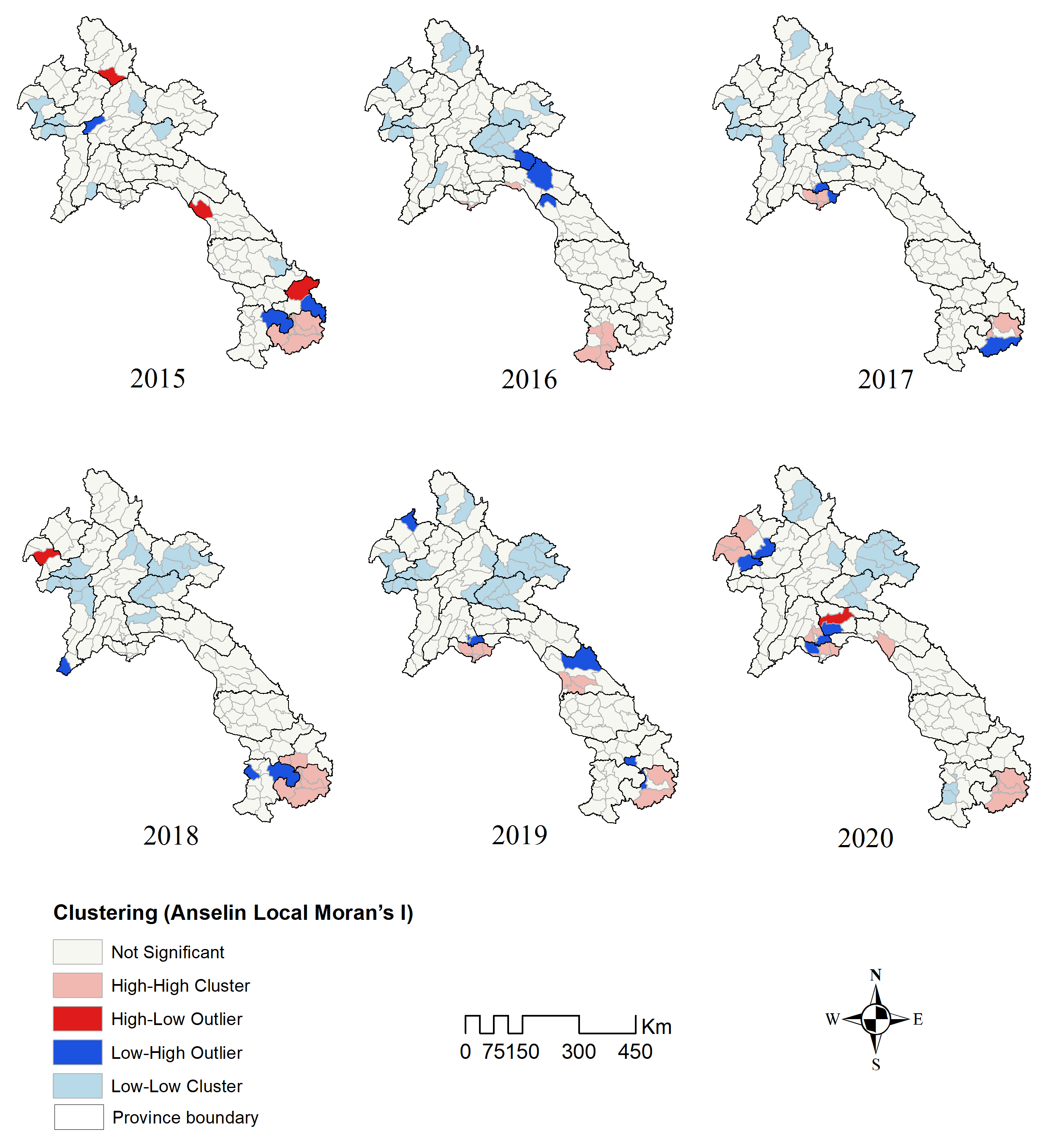

Supplement: Supplementary file 5 — Supplementary Figure S1. [file 41598_2024_71807_MOESM5_ESM.tif]
